# Data on gut metagenomes of the patients with alcoholic dependence syndrome and alcoholic liver cirrhosis

**DOI:** 10.1016/j.dib.2017.01.008

**Published:** 2017-01-17

**Authors:** Alexander V. Tyakht, Veronika B. Dubinkina, Vera Y. Odintsova, Konstantin S. Yarygin, Boris A. Kovarsky, Alexander V. Pavlenko, Dmitry S. Ischenko, Anna S. Popenko, Dmitry G. Alexeev, Anastasiya Y. Taraskina, Regina F. Nasyrova, Evgeny M. Krupitski, Nino V. Shalikiani, Igor G. Bakulin, Petr L. Shcherbakov, Lyubov O. Skorodumova, Andrei K. Larin, Elena S. Kostryukova, Rustam A. Abdulkhakov, Sayar R. Abdulkhakov, Sergey Y. Malanin, Ruzilya K. Ismagilova, Tatiana V. Grigoryeva, Elena N. Ilina, Vadim M. Govorun

**Affiliations:** a Moscow Institute of Physics and Technology, Institutskiy per. 9, Dolgoprudny, Moscow Region 141700, Russia; b Federal Research and Clinical Centre of Physical-Chemical Medicine, Malaya Pirogovskaya 1a, Moscow 119435, Russia; c Saint-Petersburg Bekhterev Psychoneurological Research Institute, Bekhtereva 3, Saint-Petersburg 192019, Russia; d Moscow Clinical Scientific Center, Shosse Entuziastov 86, Moscow 111123, Russia; e Kazan State Medical University, Butlerova 49, Kazan 420012, Russia; f Kazan Federal University, Kremlyovskaya 18, Kazan 420008, Russia

**Keywords:** Gut microbiota, Metagenome, Alcoholism, Alcohol dependence syndrome, Alcoholic liver cirrhosis, Dysbiosis, “Shotgun” metagenomics

## Abstract

Alcoholism is associated with significant changes in gut microbiota composition. Metagenomic sequencing allows to assess the altered abundance levels of bacterial taxa and genes in a culture-independent way. We collected 99 stool samples from the patients with alcoholic dependence syndrome (*n*=72) and alcoholic liver cirrhosis (*n*=27). Each of the samples was surveyed using “shotgun” (whole-genome) sequencing on SOLiD platform. The reads are deposited in the ENA (project ID: PRJEB18041).

**Specifications Table**

**Table:** 

Subject area	Biology
More specific subject area	Bacterial metagenomics
Type of data	Text files: sequences
How data was acquired	DNA sequencing using SOLiD 5500xl platform
Data format	Raw
Experimental factors	DNA extracted from stool samples
Experimental features	Barcoded fragment (non-paired) read libraries were created from 5 μg of total DNA for each of the samples. The sequencing was performed using the SOLiD 5500xl platform according to the recommendations of the manufacturer.
Data source location	Kazan, Russian Federation; Moscow, Russian Federation; Saint-Petersburg, Russian Federation
Data accessibility	The datasets are deposited in the ENA (project ID: PRJEB18041, URL: http://www.ebi.ac.uk/ena/data/view/PRJEB18041)

**Value of the data**

• The data describes the human gut microbiota composition in two cohorts of the patients with alcoholism manifesting distinct degrees of liver dysfunctions: patients with alcoholic dependence syndrome (ADS; alcoholics without advanced liver disease) and patients with alcoholic liver cirrhosis (ALC; alcoholics with advanced liver disease).
• The data can be used for identifying the changes of gut microbiota associated with alcoholism as well as with the associated liver damage at the levels of community structure (taxonomic composition) and gene potential (functional composition).
• The data can be used for validating the universality of potential biomarkers of alcoholism-associated dysbiosis via the meta-analysis together with the published gut metagenomes of the world populations.
• The data can be used in phylogeographic analyses of human microbiota to assess the genomic variations of the gut microbial species typical for Russian population as compared with the other world populations.

## 1 Data

The data represent 99 “shotgun” metagenomes of stool samples collected from the patients with ADS and ALC in 3 clinical centers from 3 Russian cities - Moscow, Kazan and Saint-Petersburg. The datasets include 25.8±16.1 mln of 50 bp reads per sample (mean±s.d., 127.5 Gbp in total). The description of the data is listed in Table 1.

## 2 Experimental design, materials and methods

### 2.1 Cohorts assembly

The study was approved by the ethical committee of the Federal Research Clinical Centre of Physical-Chemical Medicine. Each patient signed an informed consent before the start of the study. The patients were enrolled in Moscow Clinical Scientific Center (Moscow), Narcology Dispensary of Republic of Tatarstan (Kazan) and Saint-Petersburg Bekhterev Psychoneurological Research Institute (Saint-Petersburg). The cohort included 2 groups: 72 patients with the diagnosis “alcoholic dependence syndrome” and 27 - with “alcoholic liver cirrhosis”.

### 2.2 Patients inclusion and exclusion criteria

General exclusion criteria: non-alcoholic liver diseases, decompensated diseases of other organs, intake of probiotics and/or prebiotics, medications (non-steroidal anti-inflammatory drugs, antibiotics and proton pump inhibitors) less than 1 month prior to the sample collection, abdominal surgery less than 3 months prior to the sample collection. Additional inclusion criteria for the ALC group: alcoholic liver cirrhosis, age over 18 years and alcohol abuse history; the exclusion criteria were the stool changes and bowel movement frequency. Additional inclusion criteria for the ADS group: alcoholic dependence syndrome, alcohol abuse history of ≥8 years. Additional exclusion criteria specific for the ADS cohort: decrease of thrombocytes, albumin and/or prothrombin, increase of INR (international normalized ratio).

### 2.3 Sample collection and metagenomic sequencing

Stool samples were collected from the subjects, stored and subject to DNA extraction as described before [1]. “Shotgun” metagenomic libraries preparation and sequencing using SOLiD 5500xl platform (Life Technology, USA) was performed according to the recommendations of the manufacturer using the following reagent kits: 5500 SOLiD Fragment Library Core Kit, SOLiD Fragment Library Barcoding Kit, SOLiD FlowChip Kit, SOLiD FWD SR S50 Kit, SOLiD Run Cycle Buffer Kit. Barcoded fragment (non-paired) read libraries were created from 5 μg of total DNA for each of the samples. The resulting read length was 50 bp.

## Figures and Tables

**Table 1:** Information about the datasets, samples and subjects. Abbreviations: ADS - alcohol dependence syndrome, ALC - alcoholic liver cirrhosis, BMI – body-mass index, ENA – European Nucleotide Archive, NA – not available.

**Sample ID**	**ENA sample accession**	**ENA experiment ID**	**Group**	**Collection site**	**Age, years**	**Gender**	**BMI**
ADS_1	ERS1454656	ERR1748966	ADS	Saint-Petersburg	N/A	M	21.67
ADS_10	ERS1454657	ERR1748967	ADS	Saint-Petersburg	39	M	20.13
ADS_11	ERS1454658	ERR1748968	ADS	Saint-Petersburg	60	M	NA
ADS_12	ERS1454659	ERR1748969	ADS	Saint-Petersburg	41	M	20.46
ADS_13	ERS1454660	ERR1748970	ADS	Saint-Petersburg	55	M	19.86
ADS_14	ERS1454661	ERR1748971	ADS	Saint-Petersburg	29	M	23.5
ADS_15	ERS1454662	ERR1748972	ADS	Saint-Petersburg	47	M	24.76
ADS_16	ERS1454663	ERR1748973	ADS	Saint-Petersburg	43	M	20.45
ADS_17	ERS1454664	ERR1748974	ADS	Saint-Petersburg	52	M	19.35
ADS_18	ERS1454665	ERR1748975	ADS	Saint-Petersburg	41	M	21.59
ADS_19	ERS1454666	ERR1748976	ADS	Saint-Petersburg	36	M	19.23
ADS_2	ERS1454667	ERR1748977	ADS	Saint-Petersburg	50	M	21.52
ADS_20	ERS1454668	ERR1748978	ADS	Saint-Petersburg	29	M	26.8
ADS_21	ERS1454669	ERR1748979	ADS	Saint-Petersburg	45	M	24.23
ADS_22	ERS1454670	ERR1748980	ADS	Saint-Petersburg	49	M	23.04
ADS_23	ERS1454671	ERR1748981	ADS	Saint-Petersburg	26	M	23.69
ADS_24	ERS1454672	ERR1748982	ADS	Saint-Petersburg	45	M	NA
ADS_25	ERS1454673	ERR1748983	ADS	Saint-Petersburg	50	M	19.46
ADS_26	ERS1454674	ERR1748984	ADS	Saint-Petersburg	40	M	24.26
ADS_27	ERS1454675	ERR1748985	ADS	Saint-Petersburg	29	M	NA
ADS_28	ERS1454676	ERR1748986	ADS	Saint-Petersburg	52	M	NA
ADS_29	ERS1454677	ERR1748987	ADS	Saint-Petersburg	44	M	23.6
ADS_3	ERS1454678	ERR1748988	ADS	Saint-Petersburg	37	M	21.41
ADS_30	ERS1454679	ERR1748989	ADS	Saint-Petersburg	54	M	NA
ADS_31	ERS1454680	ERR1748990	ADS	Saint-Petersburg	31	M	22.6
ADS_32	ERS1454681	ERR1748991	ADS	Saint-Petersburg	49	M	NA
ADS_33	ERS1454682	ERR1748992	ADS	Saint-Petersburg	40	M	16.58
ADS_34	ERS1454683	ERR1748993	ADS	Saint-Petersburg	25	M	19.7
ADS_35	ERS1454684	ERR1748994	ADS	Saint-Petersburg	38	M	21.69
ADS_36	ERS1454685	ERR1748995	ADS	Saint-Petersburg	54	M	NA
ADS_37	ERS1454686	ERR1748996	ADS	Saint-Petersburg	32	M	17.25
ADS_38	ERS1454687	ERR1748997	ADS	Saint-Petersburg	43	M	NA
ADS_39	ERS1454688	ERR1748998	ADS	Kazan	20	M	NA
ADS_4	ERS1454689	ERR1748999	ADS	Saint-Petersburg	53	M	23.23
ADS_40	ERS1454690	ERR1749000	ADS	Kazan	30	M	21.97
ADS_41	ERS1454691	ERR1749001	ADS	Kazan	40	M	19.66
ADS_42	ERS1454692	ERR1749002	ADS	Kazan	59	F	30.76
ADS_43	ERS1454693	ERR1749003	ADS	Kazan	31	M	20.98
ADS_44	ERS1454694	ERR1749004	ADS	Kazan	60	M	25.62
ADS_45	ERS1454695	ERR1749005	ADS	Kazan	53	M	20.72
ADS_46	ERS1454696	ERR1749006	ADS	Kazan	48	M	22.49
ADS_47	ERS1454697	ERR1749007	ADS	Kazan	55	M	27.89
ADS_48	ERS1454698	ERR1749008	ADS	Kazan	45	M	21.26
ADS_49	ERS1454699	ERR1749009	ADS	Kazan	54	M	28.33
ADS_5	ERS1454700	ERR1749010	ADS	Saint-Petersburg	33	M	19.1
ADS_50	ERS1454701	ERR1749011	ADS	Kazan	36	M	21.14
ADS_51	ERS1454702	ERR1749012	ADS	Kazan	41	M	18.26
ADS_52	ERS1454703	ERR1749013	ADS	Kazan	32	M	23.6
ADS_53	ERS1454704	ERR1749014	ADS	Kazan	41	M	22.53
ADS_54	ERS1454705	ERR1749015	ADS	Kazan	34	M	28
ADS_55	ERS1454706	ERR1749016	ADS	Kazan	30	M	23.24
ADS_56	ERS1454707	ERR1749017	ADS	Kazan	59	M	24.69
ADS_57	ERS1454708	ERR1749018	ADS	Kazan	55	M	26.99
ADS_58	ERS1454709	ERR1749019	ADS	Kazan	31	M	23.46
ADS_59	ERS1454710	ERR1749020	ADS	Kazan	38	M	23.33
ADS_6	ERS1454711	ERR1749021	ADS	Saint-Petersburg	44	M	NA
ADS_60	ERS1454712	ERR1749022	ADS	Kazan	56	F	36.85
ADS_61	ERS1454713	ERR1749023	ADS	Kazan	53	F	29.74
ADS_62	ERS1454714	ERR1749024	ADS	Kazan	44	M	20.05
ADS_63	ERS1454715	ERR1749025	ADS	Kazan	52	M	23.94
ADS_64	ERS1454716	ERR1749026	ADS	Kazan	43	M	25.98
ADS_65	ERS1454717	ERR1749027	ADS	Kazan	47	M	24.54
ADS_66	ERS1454718	ERR1749028	ADS	Kazan	57	M	22.72
ADS_67	ERS1454719	ERR1749029	ADS	Kazan	57	M	26.78
ADS_68	ERS1454720	ERR1749030	ADS	Kazan	51	M	29.75
ADS_69	ERS1454721	ERR1749031	ADS	Kazan	33	M	25.47
ADS_7	ERS1454722	ERR1749032	ADS	Saint-Petersburg	37	M	21.46
ADS_70	ERS1454723	ERR1749033	ADS	Kazan	32	M	19.25
ADS_71	ERS1454724	ERR1749034	ADS	Kazan	60	F	35.49
ADS_72	ERS1454725	ERR1749035	ADS	Kazan	41	M	25.66
ADS_8	ERS1454726	ERR1749036	ADS	Saint-Petersburg	56	M	20.17
ADS_9	ERS1454727	ERR1749037	ADS	Saint-Petersburg	56	M	21.19
ALC_1	ERS1454728	ERR1749038	ALC	Moscow	46	M	33.33
ALC_10	ERS1454729	ERR1749039	ALC	Moscow	58	F	25.08
ALC_11	ERS1454730	ERR1749040	ALC	Moscow	55	M	24.49
ALC_12	ERS1454731	ERR1749041	ALC	Moscow	37	M	28.53
ALC_13	ERS1454732	ERR1749042	ALC	Moscow	32	M	37.65
ALC_14	ERS1454733	ERR1749043	ALC	Moscow	39	F	23.44
ALC_15	ERS1454734	ERR1749044	ALC	Moscow	50	F	27.34
ALC_16	ERS1454735	ERR1749045	ALC	Moscow	53	M	26.78
ALC_17	ERS1454736	ERR1749046	ALC	Moscow	54	M	30.45
ALC_18	ERS1454737	ERR1749047	ALC	Moscow	40	M	23.89
ALC_19	ERS1454738	ERR1749048	ALC	Moscow	50	M	30.02
ALC_2	ERS1454739	ERR1749049	ALC	Moscow	41	M	23.93
ALC_20	ERS1454740	ERR1749050	ALC	Moscow	52	M	20.05
ALC_21	ERS1454741	ERR1749051	ALC	Moscow	52	M	22.83
ALC_22	ERS1454742	ERR1749052	ALC	Moscow	52	M	28.39
ALC_23	ERS1454743	ERR1749053	ALC	Moscow	52	M	24.9
ALC_24	ERS1454744	ERR1749054	ALC	Moscow	54	M	26.59
ALC_25	ERS1454745	ERR1749055	ALC	Moscow	44	M	30.25
ALC_26	ERS1454746	ERR1749056	ALC	Moscow	55	M	33.91
ALC_27	ERS1454747	ERR1749057	ALC	Moscow	47	M	25.99
ALC_3	ERS1454748	ERR1749058	ALC	Moscow	57	M	25
ALC_4	ERS1454749	ERR1749059	ALC	Moscow	52	F	22.77
ALC_5	ERS1454750	ERR1749060	ALC	Moscow	58	M	30.93
ALC_6	ERS1454751	ERR1749061	ALC	Moscow	49	F	27.06
ALC_7	ERS1454752	ERR1749062	ALC	Moscow	42	M	31.83
ALC_8	ERS1454753	ERR1749063	ALC	Moscow	57	M	24.07
ALC_9	ERS1454754	ERR1749064	ALC	Moscow	44	M	30.72
